# Non-oral pharmacological interventions in the management of herpes zoster-related pain: a review of current research

**DOI:** 10.3389/fpain.2024.1485113

**Published:** 2024-11-27

**Authors:** Yaojun Wang, Yanxia Shen, Haixue Guo, Dongcai You, Shimin Jia, Ge Song, Xiaobing You

**Affiliations:** ^1^ Clinical Medical College, Hebei University, Baoding, Hebei, China; ^2^ Pain Department, The Second Hospital of Handan, Handan, Hebei, China; ^3^ Infirmary, Handan Vocational College of Technology, Handan, Hebei, China

**Keywords:** herpes zoster, postherpetic neuralgia, neuropathic pain, intervention, interventional therapy

## Abstract

Herpes zoster-associated pain is a difficult-to-treat pathologic pain that seriously affects patients' quality of life. In recent years, emerging therapeutic techniques such as autologous platelet-rich plasma, sympathetic nerve block and pulsed radiofrequency have been gradually applied in the field of pain with the advantages of less trauma, quicker recovery and significant efficacy. These therapeutic options have become a new hope for the treatment of herpes zoster-associated pain. This article reviews the studies on herpes zoster-associated pain in non-oral drug therapy, summarizes the efficacy, safety, and possible mechanisms, and provides a reference basis for clinical treatment.

## Introduction

Zoster-Related Pain (ZRP) is pain caused by Varicella-Zoster Virus (VZV) infection that significantly affects the quality of life of patients (1). More than 90% of the world's population is infected with VZV during childhood, and as specific immunity wanes, the virus can reactivate, leading to herpes zoster and associated pain (2). ZRP is categorized into Acute Herpes Zoster-Related Pain (AHZRP), Subacute Herpes Zoster-Related Pain and Chronic Herpes Zoster-Related Pain (CHZRP). AHZRP is characterized by a sharp stabbing and burning sensation, often described by patients as a “cutting” or “electric shock” pain, limited to the area of the attack and distributed along the affected nerve roots, usually starting a few days before the rash appears. The pain is confined to the area of the attack and distributed along the affected nerve roots, usually starting a few days before the rash appears (3). CHZRP, called postherpetic neuralgia (PHN), can last for months or even years and is characterized by allodynia, persistent burning sensation, intense itching, hyperalgesia, and tactile hypersensitivity, which complicates the management of PHN (1). Visual analog scale (VAS), numeric rating scale (NRS), verbal rating scale (VRS) and facial expression pain scale (FPS) are mostly used to assess pain intensity.

During an acute episode of herpes zoster, the goal of treatment is to control symptoms and prevent complications, and commonly used treatments include antiviral therapies, corticosteroids, and analgesics. Antiviral drugs (e.g., acyclovir, valacyclovir, and famciclovir) accelerate rash healing and reduce pain levels by inhibiting VZV replication and shortening the viral shedding period (4, 5). However, the antiviral effect is significantly reduced if the rash is not treated promptly within 72 hours of rash appearance. Corticosteroids may be used in combination with antivirals to reduce acute pain, but should not be used alone and do not reduce the incidence of PHN (6). Acute pain usually requires additional analgesics, NSAIDs are often insufficiently effective, and opioids may be considered (7). Treatment of PHN is extremely challenging, and in addition to traditional medications, first- and second-line pharmacological therapies (e.g., tricyclic antidepressants, 5-hydroxytryptamine-norepinephrine reuptake inhibitors, pregabalin, gabapentin, and tramadol, among others) are recognized as having grade A evidence of pain relief (8, 9). Effects remain limited (8, 9). Even when multiple oral medications are combined, pain relief is often incomplete, highlighting the complexity of PHN management.

ZRP causes significant suffering to patients, and conventional oral drug therapy has limitations and adverse effects. Antiviral drugs (e.g., acyclovir, vasiclovir) inhibit VZV but do not reduce the incidence of PHN, and can also cause nausea, neurotoxicity, and renal damage (10). Opioids, on the other hand, carry the risk of addiction and respiratory depression, and corticosteroids may lead to metabolic disorders and mood swings, and are usually only indicated for those with severe symptoms or without contraindications (11). Therefore, finding more effective and safer treatments has become a focus of research. In this paper, we review the research progress of non-oral drug treatment of ZRP, focusing on summarizing its action, safety and mechanism to provide clinical reference.

## 2 Epidural or intrathecal injection of cortisol hormone

Histopathologic studies in patients with PHN have shown that infiltration of lymphocytes occurs in the posterior horn of the spinal cord during subacute and chronic inflammatory processes, suggesting that inflammation may play an important role in the development and progression of PHN (12). In addition, the concentration of interleukin-8 (IL-8) in the cerebrospinal fluid of patients with PHN is significantly elevated, and studies have confirmed that IL-8 is associated with pain triggered by inflammatory responses (13).PHN causes intense spinal inflammation that persists for several years before gradually resolving, and the concentration of IL-8 is inversely correlated with the duration of PHN before treatment (14). Given the potent anti-inflammatory effects of corticosteroids, it is able to reduce nerve damage and thus alleviate the pain of PHN (15). Intrathecal injection of methylprednisolone further supports its potential anti-inflammatory effect by reducing IL-8 concentrations (14). A randomized controlled trial comparing intrathecal midazolam and epidural methylprednisolone showed that epidural methylprednisolone had a long-term analgesic effect in lumbosacral dermatomal PHN (16). In addition, in a study by Dureja G P et al, a single dose of intrathecal midazolam combined with epidural methylprednisolone reduced AHZAP but did not prevent PHN (17). Another matched cohort study of 427 participants also showed that corticosteroids helped to relieve the acute phase of infection but were not effective in preventing PHN (18). A small randomized controlled study found no significant difference in patients with postherpetic neuralgia with intrathecal injection of methylprednisolone acetate compared to controls (19). The effectiveness of epidural or intrathecal cortisol hormone injections for ZRP remains controversial. In addition, the side effects of epidural injection of cortisol hormone, such as chemical meningitis, transverse myelitis, cauda equina syndrome, lumbar radiculitis, intractable headache, and urinary retention should not be ignored (20). Therefore, although epidural anesthetics and steroids can alleviate PHN (15), more clinicians are needed to weigh the benefits of this treatment.

## 3 Botulinum toxin type A (BTX-A)

BTX-A injection, an emerging therapy for ZRP treatment, has shown significant pain relief (21, 22). The mechanism of action may include reducing neurogenic inflammation and preventing peripheral sensitization (23). Studies have shown that BTX-A inhibits the release of neurotransmitters such as acetylcholine, glutamate, substance P, and calcitonin gene-related peptide, thereby reducing neuropathic pain (24). Based on these theories, BTX-A is gradually being used in ZRP. Recent randomized controlled trials have shown that BTX-A injections resulted in significant reductions in VAS scores in painful areas, prolonged sleep, and reduced opioid use (25–27). In one study, all 13 patients with PHN had a decrease in their VAS scores after receiving BTX-A injections for 2 weeks (28). A preliminary study by Freund and Schwartz showed a decrease in VAS scores from 8/10 to 5/10 in 7 patients with PHN, although the lack of a control group failed to draw definitive conclusions (29). In addition, an 80-year-old patient experienced significant pain relief with multiple BTX-A injections for 52 days after conventional treatment failed (30). Despite the favorable efficacy of BTX-A, it may also lead to adverse effects such as local muscle weakness, discomfort at the injection site, and systemic allergic reactions (31). Therefore, when BTX-A is applied to treat PHN, the health status of the patient should be adequately assessed and monitored after treatment to reduce the risk of adverse reactions. In conclusion, although BTX-A has demonstrated positive effects in ZRP treatment, rigorous evaluation before injection remains crucial.

## 4 Platelet-rich plasma (PRP)

PRP is activated by calcium chloride and thrombin to release a variety of growth factors that support nerve repair and serve as a clinical adjunctive therapeutic tool (32). The pathogenesis of ZRP is related to inflammation and nerve damage caused by viral replication, and inflammation can sustain damage to peripheral and central neurons and enhance pain sensitivity (33). Therefore, controlling the inflammatory response to promote nerve repair is key to the treatment of ZRP. Growth factors in PRP modulate inflammation and promote tissue regeneration (34–36). It also increases the secretion of platelet microbial protein (PMP), which significantly reduces inflammation (37). In addition, IGF-1 and VEGF factors in PRP promote spinal cord axon proliferation and restore innervation (38) Studies have shown that PRP is associated with the proliferation and neurophysiological activity of rat chervon cells, which play an important role in the maintenance and regeneration of peripheral nerves (39). Thus, PRP may exhibit ZRP therapeutic potential by reducing inflammation and promoting nerve regeneration in peripheral nerves. Studies on PRP in ZRP prevention and treatment are still limited. Zhou Z et al. (40) showed that in the observation group, an ultrasound-guided local paravertebral nerve puncture technique was used to inject 1 ml of PRP into each nerve segment. The results showed that the NRS score in the observation group was significantly lower than that in the control group, which was accompanied by improved sleep quality, reduced drug use, and shorter time for herpes to dry up and scab over. In addition, the incidence of dizziness, drowsiness, ataxia and PHN in the observation group was also significantly reduced. Despite the positive results of PRP in ZRP treatment, localized adverse effects such as pain, swelling and bruising at the injection site may occur, and individual patients may be at risk of systemic allergy or infection. Overall, PRP has a high safety profile, but clinical application requires proper evaluation and monitoring to identify potential adverse reactions.

## 5 Sympathetic nerve block

### 5.1 Epidural nerve block (ENB)

In clinical practice, ENB is commonly used to achieve sustained pain relief in patients with herpes zoster pain in whom oral medications are ineffective. Despite the lack of objective evidence, continuous epidural block is thought to relieve PHN and shorten the duration of acute herpes zoster treatment and reduce the incidence of PHN (18, 41). A clinical trial showed that patients who received epidural bupivacaine in combination with methylprednisolone had a 1-year pain incidence of only 1.6%, which was significantly lower than that of the control group, which was 22.2% (18). In addition, continuous epidural nerve blocks have been shown to be effective in the long-term control of chronic neuropathic pain (42, 43). In a case report, a 69-year-old patient with PHN who had failed to respond to oral treatment obtained relief with repeated injections of local anesthetic in the paravertebral space (44). A retrospective study by Manabe H et al. noted that continuous epidural nerve block combined with antiviral medication significantly shortened the duration of treatment for AHZRP and resulted in a more rapid reduction in pain intensity without associated complications (45). A study by Pasqualucci A et al. also showed that epidural nerve block combined with methylprednisolone was superior to intravenous acyclovir combined with prednisolone in relieving PHN (18). Systematic evaluations and meta-analyses have further recommended the application of epidural nerve blocks in the acute phase of herpes zoster to shorten the duration of ZRP and prevent PHN (46, 47). Despite the effectiveness of ENB in reducing pain, its common adverse effects include pain at the injection site, bleeding, and infection, which may lead to serious complications such as nerve damage, conduction disturbances, or spinal abscesses. Patients may also experience systemic reactions such as hypotension, headache or low back pain (46). Therefore, clinical application requires careful assessment of indications and contraindications and monitoring of post-treatment response for individualized management.

### 5.2 Stellate ganglion block (SGB)

The sympathetic nervous system is considered an important mediator of pain (48). Following nerve injury or tissue inflammation, lateral sprouting and upregulation of functional adrenergic receptors in peripheral and dorsal root ganglia may lead to the formation of anatomical and chemical coupling between sympathetic postganglionic neurons and afferent neurons. Also, sympathetic nerve endings may increase the sensitivity of injurious afferent nerves (49). Although the mechanism of action of the sympathetic nervous system in PHN is uncertain, studies have shown that stellate ganglion block (SGB) improves the tissue environment by blocking sympathetic conduction, promoting vasodilatation in the upper extremities, upper thoracic segments, and head and face, increasing local blood flow, and removing inflammatory substances and metabolites (50). In addition, SGB can reduce the levels of pain inflammatory factors and mediators in the plasma of ZRP patients, enhance immune cell activity, promote nerve repair and regeneration, and effectively relieve pain symptoms (48). In clinical practice, a 65-year-old patient with recalcitrant AHZRP was treated with SGB, which significantly relieved pain in the head, face, and upper extremities (51). A study by Makharita M Y et al. demonstrated that SGB combined with antiviral medication significantly reduced the intensity and duration of AHZRP and reduced the incidence of PHN (52). Case reports have also confirmed the effectiveness of SGB in patients with herpetic neuralgia, demonstrating a significant reduction in VAS scores (53, 54). However, serious complications and even death may occur after SGB (55). Common adverse reactions include discomfort, swelling, and bruising at the local injection site. Patients may also experience drowsiness, dry eye, pupil changes, or hoarseness, all due to effects on the autonomic nervous system. In rare cases, SGB may cause serious complications such as pneumothorax or hematoma (56). Therefore, patients should be thoroughly evaluated when performing SGB, and appropriate postoperative monitoring should be performed to minimize risks and ensure safe and effective treatment.

### 5.3 Paravertebral block, (PVB)

PVB can be used as an alternative therapy to epidural nerve block for short-term relief of recalcitrant PHN (57). PVB is a drug injection technique that produces a unilateral motor, sensory, and sympathetic blockade effect by injecting drugs adjacent to the intervertebral foramen of a spinal nerve (44). This technique reduces peripheral pain and afferent signals by blocking sensory nerves, promotes vasodilatation in the lesion area, and improves local circulation in order to eliminate the vicious cycle of pain response. Several randomized controlled trials have shown that PVB is effective in relieving ZRP, reducing the incidence of PHN, as well as reducing oral drug doses and improving sleep quality compared to antiviral therapy alone (58, 59). The results of a randomized controlled trial showed that patients who received peripheral nerve block (ESB) and PVB had significantly lower pain NRS scores than controls and had significant relief of both AHZRP and persistent PHN at 6 months (60). In addition, a study showed that repeated paravertebral blocks with local anesthetics and steroids provided safe and effective pain relief and reduced the incidence of PHN in patients with acute thoracodorsal herpes zoster (61). In another case report, a 72-year-old man with abdominal segmental hernia, constipation, and PHN received significant pain relief after PVB treatment (62). In addition, a retrospective study by Xue M and Yuan R noted that the ultrasound-guided peripheral nerve blocks (ICNBs) technique in the epidural space is simpler and less time-consuming compared to the traditional technique of transparietal perforator puncture epidural blocks (TPVBs), and may become a more accessible means of preventing PHNs (63). Adverse effects of PVB are less frequent and mainly include injection site pain, swelling and bruising, and individual patients may experience hypotension or changes in heart rate, especially when large amounts of anesthetic drugs are used. Therefore, PVB can be an effective intervention for ZAP while ensuring safety.

## 6 Pulsed radio frequency (PRF)

PRF is a minimally invasive, targeted therapeutic technique that controls heat below 42°C (64) by pulsed current to avoid nerve damage (65) and is commonly used to alleviate PHN (66). It has been found that patients with ZRP have degenerative changes in class C fibers, which may lead to reorganization of pain signaling pathways in the central nervous system, activation of fiber reflexes, and lowering of the pain threshold, which in turn exacerbates neuropathic pain (67). The potential mechanism of PRF is that a rapidly changing electric field acts on the neuronal cell membranes, inducing electrolyte conduction and depolarization, which reduces pain and protects the nerves (64). Currently, PRF is mainly used in the treatment of pain in dorsal root ganglia, spinal ganglia and sympathetic ganglia. A randomized triple-blind controlled trial showed that PRF was superior to local anesthetic drug injections in the treatment of radicular pain and reduced TNF-α concentrations and CD3^+^ counts in cerebrospinal fluid (68). Another retrospective study showed that patients' VAS scores, Pittsburgh Sleep Quality Index (PSQI) and 36-item Brief Health Survey (SF-36) scores were significantly lower after single vs. two PRF treatments, and that the overall effectiveness rate was higher in the two-treatment group than in the single-treatment group. In addition, several reports have confirmed the effectiveness of PRF in controlling pain in patients with PHN and acute herpes zoster (69–71). PRF likewise provides significant relief of recalcitrant ZRP pain (69, 72). Studies have shown that VAS scores 6 months after PRF were significantly lower than those of the control group, and SF-36 scores improved significantly on all functional and mental health measures (69). A meta-analysis also showed that PRF significantly relieved ZRP pain and was associated with only minor adverse events, such as localized symptoms and transient bradycardia (73). In summary, PRF is a minimally invasive, targeted treatment technique that avoids nerve damage by controlling heat, can both effectively relieve ZRP, and shows good efficacy and relatively few adverse events.

## 7 Spinal cord stimulation (SCS)

The mechanism of SCS in the treatment of ZRP is unclear, but the rationale for spinal cord electrical stimulation in pain modulation is supported by the “gate control” theory, in which neural signaling is regulated by the dorsal horn of the spinal cord (74). SCS may reduce neuropathic pain by affecting the levels of γ-aminobutyric acid and adenosine in the dorsal horn (75). This method uses electrical current to stimulate spinal cord nerve fibers by implanting electrodes at specific locations in the spinal cord, altering the transmission and perception of pain signals (76) and modulating nerve fiber excitability for further pain relief (77). Several studies have demonstrated that SCS is an effective treatment for recalcitrant PHN (78–80). Early application of SCS significantly relieves PHN pain (81), with relief rates ranging from 27% to 82% (82). Small randomized controlled double-blind trials have shown that both PRF and SCS are effective alternative therapies for acute/subacute herpes zoster-related pain, but SCS achieves more significant pain relief and quality-of-life improvement than PRF (83). A longitudinal study of 28 patients by Harke H and Gretenkort P et al. demonstrated that 23 patients with postherpetic neuralgia and 4 acute patients with chronic pain improved after electrical stimulation (78). In addition, temporary SCS also reduced subacute herpetic pain and prevented the development of chronic pain (84, 85). A retrospective study showed that among 32 ZRP patients treated with SCS, VAS scores decreased significantly, 18 patients (39.1%) achieved complete pain relief, and no serious adverse effects were observed throughout the follow-up period (86). SCS therapy consists primarily of short-term spinal cord stimulation (stSCS) and permanent spinal cord stimulation. The effectiveness of temporary electrodes needs to be tested initially before implanting permanent electrodes. If temporary SCS is effective, permanent electrodes can be implanted after several weeks (87). Although permanent SCS can provide long-term analgesia, its application faces the complexity of patient selection and high cost. In contrast, stSCS has received increasing attention due to its simplicity, low cost and high efficiency. Compared with conventional SCS, temporary SCS is less costly and less invasive (88). A Japanese study showed that temporary SCS was effective in reducing PHN and preventing its occurrence (77). In addition, SCS combined with microsurgical posterior rhizotomy also improves the treatment outcome of PHN (82). However, SCS still has some drawbacks such as e.g., dislocated electrodes, broken wires or malfunctioning stimulators. In addition, patients may feel abnormal, tingling or numbness. In summary, despite the potential application of SCS in ZRP therapy, patients should be adequately evaluated preoperatively and monitored postoperatively during implementation, and further research and clinical practice will help to explore the potential of SCS in ZRP therapy and provide more effective pain management programs for patients.

## 8 Other methods

The pathophysiology of ZRP encompasses both central and peripheral mechanisms, resulting in complex and varied treatment. Peripheral electrical nerve stimulation, as a highly targeted and less invasive form of neuromodulation, is commonly used to treat patients with intractable neuropathic pain. This technique modulates nerve fiber excitability through continuous stimulation of peripheral nerves and alters pain signaling pathways to relieve pain (89). The results of several small studies have shown that patients treated with peripheral neuromodulation significantly reduced their pain symptoms over a follow-up period of more than 6 months (90–92). These studies support the potential value of peripheral nerve electrical stimulation in improving chronic pain management.Studies by Green A L et al. have shown that deep brain electrical stimulation of the gray matter around the contralateral ventricles and the ventral posterolateral part of the thalamus can significantly reduce the symptoms of post-herpes zoster neuralgia (PHN).In this study, a patient with sensory impairment on the right side of the face caused by herpes zoster infection experienced pain for up to 10 years. After a period of 6 months of electrical stimulation treatment, the final follow-up VAS score was 0/10, showing a significant therapeutic effect (93).

In addition, a retrospective study showed that transcutaneous electrical nerve stimulation has a positive effect in preventing the development of PHN.The work of Stepanović A and other investigators confirmed the effectiveness of transcutaneous electrical nerve stimulation in reducing the incidence of subacute herpetic neuralgia (94). According to a report by Kolšek et al, transcutaneous electrical nerve stimulation was more effective in reducing and preventing PHN compared to conventional antiviral drugs, suggesting the importance of this method in clinical application (95). Meanwhile, another study noted that the combination of transcutaneous electrical nerve stimulation combined with methylcobalamin demonstrated a favorable analgesic effect on PHN (96).

## 9 Summary

Significant progress has been made in the treatment of ZRP with the continued advancement of non-oral drug therapy techniques. However, when selecting an appropriate treatment regimen, the safety and relevance of the treatment must be prioritized. Current research suggests that nonoral treatments such as subcutaneous injection of botulinum toxin A or tretinoin, transcutaneous electrical nerve stimulation, peripheral nerve stimulation, and stellate ganglion block may be the preferred option for those patients who do not respond well to oral medications. In addition, there are significant differences in the pathomechanisms of acute pain and postherpetic neuralgia that occur during active herpes zoster. Acute pain is usually associated with an active inflammatory response, whereas PHN is associated with chronic pain mechanisms such as nerve remodeling and increased central sensitization. Therefore, the patient's pain type should be specifically analyzed when developing a treatment plan. Therapies such as paravertebral blocks and pulsed radiofrequency have also shown some potential in acute herpes zoster pain, however, if the pain is severely persistent, spinal cord electrical stimulation should be considered at this time.

In addition, these minimally invasive interventional techniques have a wide range of applications not only in ZAP, but also for the effective treatment of other pathologic neuropathic pain. For example, SCS and peripheral nerve stimulation have shown significant pain relief in patients with diabetic neuropathy. SGB and PRF techniques also play an important role in complex regional pain syndromes by blocking sympathetic nerves and modulating neural activity to alleviate chronic pain.PVB and ENB are also widely used in postoperative pain control, effectively reducing patients' pain sensations and opioid requirements. In addition, PRF and PVB are able to relieve radicular pain caused by disc herniation by targeting the nerves. In summary, although these approaches can provide pain relief for patients, they need to be implemented with caution, especially when considering the destructive nature of dorsal root ganglia and the potential adverse events of intrathecal methylprednisolone injections. Therefore, larger, multicenter clinical trials are urgently needed to clarify the exact efficacy and side effects of these minimally invasive interventional techniques and to explore their applicability and mechanisms in the management of other virus-induced neuropathies.
